# Editorial: Pediatric cardiology and cardiac surgery in developing countries: Current needs and future perspectives

**DOI:** 10.3389/fped.2022.1067193

**Published:** 2022-11-25

**Authors:** Alessandro Giamberti, Mauro Lo Rito, Giovanni Stellin, Tom Karl, Alessandro Frigiola

**Affiliations:** ^1^ Bambini Cardiopatici nel Mondo Association NGO, Milano, Italy; ^2^ Department of Pediatric and Adult Congenital Cardiac Surgery, IRCCS Policlinico San Donato, Milano, Italy; ^3^ Pediatric and Congenital Cardiac Surgery Unit, Department of Cardiac, Thoracic and Vascular Sciences and Public Health, University of Padua, Padua, Italy; ^4^ Queensland Pediatric Cardiac Service, Queensland Children’s Hospital, Brisbane, Australia

**Keywords:** pediatric cardiac surgery, developing countries, international cooperation, congenital heart disease, humanitarian medicine

**Editorial on the Research Topic** Pediatric cardiology and cardiac surgery in developing countries: Current needs and future perspectives By Giamberti A, Lo Rito M, Stellin G, Karl T and Frigiola A. (2022). Front. Pediatr. 10:1067193. doi: 10.3389/fped.2022.1067193

Cardiovascular diseases are the leading cause of death worldwide and, despite the progress of medicine in recent decades, 17.5 million deaths annually occur due to these non-communicable diseases (1). Cardiovascular diseases can be acquired or congenital, the first affects elders, while the second occurs at birth because they are a defect of the heart formation. Congenital heart disease (CHD) reduces the survival expectancy, quality of life, and work capacity of patients, but also affects the entire family and society. The WHO estimates that approximately 1.5 million newborns each year are affected by CHD, and more than 4 million children are waiting for cardiac surgery treatment worldwide (2). In addition, in low- and middle-income countries (LMICs), the high prevalence of rheumatic heart disease (RHD) increases the number of children who require cardiological care. Today, due to the progress of pediatric cardiology and cardiac surgery, most patients born with CHD can be diagnosed prenatally and treated at the appropriate age. Such medical progress significantly reduces the mortality burden, allowing more than 90% of children with CHD to reach adulthood in Western Europe or North America with a good quality of life. Unfortunately, in LMICs, a significant proportion of children with CHD are destined to die because they receive suboptimal care or do not have access to medical attention (2–4).

The dramatic disproportion of medical access and healthcare services between developed countries and LMICs is particularly evident in the treatment of CHD (3). More than 70% of healthcare facilities are accessible only to less than 20% of the world's population. Moreover, it is not guaranteed that such expensive structured programs (congenital cardiology and cardiac surgery) will receive regular economic support. Several programs, projects, and collaborations have been developed in recent decades to fill these gaps, providing valuable examples of successful and unsuccessful ideas. But nowadays, with the global economic crisis and the COVID-19 pandemic, the future of such a battle against inequality is even more uncertain and complex.

The purpose of the Research Topic *“Pediatric Cardiology and Cardiac Surgery in Developing Countries: Current Needs and Future Perspectives”* is to bring together experts around the world to share their experiences and evaluate the participation of leading actors in humanitarian medicine. We aim to depict a broad picture of successful programs and assess possible solutions and future developments.

Fourteen original research articles have been selected in this special issue that covers four major themes:

1. Cooperation models
2. Epidemiology and risk factors in CHD
3. Education in pediatric cardiology and cardiac surgery
4. Excellence in CHD care.

## Cooperation models

There are different cooperative models to support the treatment of children with CHD from LMICs, and all have limitations (1, 2, 4).

A frequently adopted model is based on the transfer of CHD patients from LMICs to experienced centers in developed countries for treatment. This cooperation model carries several limitations, such as the cost of expenses for the families, administrative barriers (i.e., VISA permits), and, more importantly, the fact that a few lucky patients can be treated in such a way.

The second model is based on surgical missions overseas from expert teams in a selected local LMIC institution. This is the most widespread model but heavily relies on financial resources and trained caregivers' availability.

The third model is based on the establishment of a long-term collaboration between a local hospital and a well-known, experienced international partner to create stable and autonomous local services. This model requires great financial support for local staff training and geopolitical stability to succeed. Still, it guarantees the creation of durable healthcare services and good long-term results.

In this special issue, different successful models have been described by their strengths and weaknesses, particularly how they are affected by global socio-economic conditions.

Giamberti et al. quantify the impact of the COVID-19 pandemic on the activity of Italian congenital cardiac surgery centers in their respective charitable programs. They compare data from humanitarian activities carried out abroad or on-site from 2019 (pre-COVID-19) to 2020 (COVID-19 pandemic). The pandemic led to a reduction in the activity of 96% of procedures performed overseas and 86% of surgeries performed in Italy. The authors show how receiving congenital cardiac patients from LMICs and completing surgical missions abroad are still the most used models of cooperation in Italy. The incredible impact of the pandemic highlights the failure of these cooperation methods that do not have sufficient resilience to major constraints. A completely different approach is suggested through the creation of a worldwide international body and scientific society networks that should play a leadership role in coordinating and organizing such efforts.

Marianeschi et al. review the experience of an important Italian NGO, “Mission Bambini”, founded in 2000, and the results they obtained when they launched the “Children's Heart Program” based on long-term partnerships to provide multidisciplinary education and training and technical support for cardiac surgery programs in LMICs. In 2010, the NGO started changing policy, switching from being a donor foundation to being an implementer foundation taking charge of the ideation and execution of its programs dedicated to CHD patients in a few selected local hospitals.

Marianeschi et al. also propose an interesting way to assess the socio-economic impact of humanitarian missions. Using a qualitative and quantitative parameters score called DALY (Disability Adjusted Life Years), they evaluate the socio-economic effects of their cooperation model in a single project. The DALY measures the disease burden in terms of years of life and year of disability lost and regained after CHD was repaired. They applied the DALY score to 128 congenital cardiac patients operated on between 2012 and 2019 in Cambodia, showing that surgery for CHD allows patients to regain quality of life, life expectancy, and socio-economic roles at a very low cost.

This section ends with the experience of two cardiac surgery centers in Africa, created in collaboration with some Italian NGOs.

In the first article, Langer et al. report the activity of one of the most important Italian NGOs called EMERGENCY. EMERGENCY is well known worldwide; it was founded in 1994 with the primary goal to provide life-saving surgery for war-wounded people by building hospitals in some of the countries most covered in landmines and devastated by conflicts. In 2007, they created the Salam Center for Cardiac Surgery in Sudan to offer a stable cardiological and surgical service in such African regions. Since its inauguration, more than 8,000 patients have undergone open heart surgery completely free of charge, thanks to the support of the Sudanese government. The authors report excellent results, not only for CHD but also for valve surgery in 1,318 children suffering from RHD. We were delighted to read this article that informs the medical community about this extraordinarily successful project. The Salam Center in Sudan offers a model of cooperation that should be replicated with all the necessary ingredients: high-quality medicine for all, totally free of charge, continuity, affordability, sustainability, and complete autonomy.

Finally, the article by Mvondo et al. describes another example of international humanitarian cooperation between two Italian NGOs (Bambini Cardiopatici nel Mondo Association and Cuore Fratello Association) and the religious order Tertiary Sisters of St. Francis to open the first Cardiac Center of Cameroon in the North-West Province of Shisong. The article highlights a challenging period characterized by an important local socio-economic crisis, and the pandemic represented a further challenge toward autonomy and sustainability. With great efforts, they have transferred part of the medical equipment and human resources to the capital Yaoundè. After two years of complete activity stop, they slowly resumed full autonomy. All our admiration and encouragement go to them in continuing the difficult path.

## Epidemiology and risk factors in CHD

The first article selected in this section is a fascinating study from South Africa (Adersley et al.) describing the PROTEA project. The PROTEA project aims to establish a cohort of phenotyped and genotyped CHDs in their region to facilitate research on the epidemiology and genetic determinants of CHD. The initial cohort of 1,473 patients was recruited from seven hospitals in the Western Cape province (April 2017–March 2019). One of the important findings is the different incidence of CHD compared to other international studies on global birth prevalence. The study shows a reduced incidence of simple and mild forms of CHD compared to literature references. These significant findings suggest that mild CHDs which are the most curable and have the best long-term survival remain undiagnosed in South Africa.

The article from Arnaiz et al. analyzes 897 children aged 8–16 years from 8 peri-urban schools in the Eastern Cape of South Africa. The purpose of the study was to evaluate the incidence of arterial hypertension and compare their data with international references. The authors find that international standards may be inadequate because they have often been developed in populations of different continents. They advocate for the development of normative tables that should be more representative of the South African pediatric population.

Shao-Ju Chien et al. from Taiwan present a pilot study that analyzes the Taiwan National Health Insurance Research Database to evaluate the fertility of Taiwanese women with a diagnosis of CHD and how different forms of CHD may have influenced fertility in various ways. The results show that CHD compromises female fertility, even among patients with simple forms of CHD. They suggest that pregnant women with CHD should be managed by a multidisciplinary team, including pediatric cardiologists, obstetricians, and anesthetists with relevant experience.

The last article in this section by Hao Chen et al. is from the Shangai Children's Medical Center of China. They analyze the outcome of 1,078 newborns with CHD who underwent cardiac surgery over 15 years (2006–2019). Despite a global improvement in the results in the last period, mortality remains elevated. The authors identify four risk factors for mortality: two non-modifiable (low weight and high complexity CHD) and two modifiable (poor pre-operative health status and surgeon experience). They advocate for improving prenatal diagnosis, increasing pediatric cardiologists in community hospitals, and developing better transfer systems for these patients in China.

## Education in pediatric cardiology and cardiac surgery

In this section, we present two interesting studies that can improve and facilitate clinical and diagnostic practice, especially when faced with complex cardiological problems in low-resource settings.

In the first article from Indonesia, Rahmat et al. aim to define which patients with atrial septal defects have a borderline left ventricle and may experience a difficult and costly post-operative course. They find that the left end-diastolic volume indexed (LVEDVi) on an MRI scan was the most important parameter associated with postoperative low cardiac output syndrome in adult patients with atrial septal defects and small left ventricles. An LVEDVi less than 53.5 ml/m^2^ is suggested to be the best predictor of adverse outcomes in these patients.

The second article by Sabatino et al*.* is an international multicenter study aimed at demonstrating the feasibility of noninvasive myocardial work (MW) parameters in the pediatric population. Non-invasive MW parameters have already highlighted their effectiveness in different studies regarding children with Turner syndrome and Kawasaki disease. The article shows the feasibility of non-invasive MW in a healthy pediatric population and provides useful two-dimensional normative reference ranges for MW parameters not influenced by age, gender, and BW and can be largely applied with a particular interest in LMICs.

## Excellence in CHD care

The last section includes three articles that highlight how excellent results can be obtained in the surgical treatment of the more complex CHD in LMICs.

The first study from China (Qi Lou et al.) analyzes 10 years of experience in the surgical treatment of Ebstein's anomaly and a large volume of patients (170). They compare two surgical techniques for the repair of the Ebstein tricuspid valves in a large contemporary cohort. They compare the Cone and Hetzer repair, adding further scientific support to the Cone technique, considered today the gold standard in the surgical treatment of this complex congenital pathology.

Ta A. Tuan et al, from Hanoi, Vietnam, present one of the most extensive series in an LMIC using ECMO support in acute myocarditis. They show excellent results with a low incidence of strong neurological complications. Finally, Bezerra et al, from Sao Paulo, a reference center in Brazil, report the results of ECMO support for neonates with hypoplastic left heart syndrome submitted to the Norwood procedure. Over 5 years (2015–2019), they performed 120 Norwood procedures. ECMO support was used in 28% of the patients with a survival rate close to 50%.

The United Nations Development Program's (UNDP) sustainable development goal for 2030 is to reduce mortality to 25 per 1,000 live births for children under 5 years of age. To achieve such a goal, the care of CHD and RHD needs to be part of this strategic plan, considering that heart disease is among the most frequent causes of death in this age group, following malnutrition and infections.

Today, international cooperation through NGOs is experiencing a progressive decline related to the global economic crisis, the COVID-19 pandemic, the reduction in funding and volunteers, and the frequent unreliability of local partners.

A global strategy is needed to improve cardiovascular delivery care in poor countries and maximum effort should be made for a better future.
